# Programming for Meiotic Competence in Mouse Male Germ Cells is Established at the Perinatal Precursor Stage of Development

**DOI:** 10.1002/mrd.70032

**Published:** 2025-05-25

**Authors:** Qi‐En Yang, Mingyao Yang, Melissa J. Oatley, Jon M. Oatley

**Affiliations:** ^1^ Center for Reproductive Biology, School of Molecular Biosciences, College of Veterinary Medicine Washington State University Pullman Washington USA; ^2^ Key Laboratory of Adaptation and Evolution of Plateau Biota, Northwest Institute of Plateau Biology Chinese Academy of Sciences Xining Qinghai China

**Keywords:** meiosis, programming, prospermatogonia, spermatocyte

## Abstract

Meiosis is a fundamental aspect of gametogenesis, but how and when the programming is established in germ cells during development is unknown. In the mammalian male germline, mitotic differentiating spermatogonia with the competence for meiotic divisions arise from an undifferentiated pool of spermatogonia that are descended from prospermatogonial precursors. Here we provide evidence from mouse models that suggests programming for meiotic competence is established much earlier in the developmental trajectory of spermatogonia than previously believed, likely at the prospermatogonial stage in fetal life. Conditional overexpression of the gene *Id4* in prospermatogonia led to a block in meiotic progression of spermatocytes during postnatal spermatogenesis. In contrast, meiotic progression was found to proceed when *Id4* was conditionally overexpressed beginning in postnatal spermatogonia. Moreover, conditional overexpression of *Id4* in the female germline beginning at the fetal stage of development after oocytes have initiated meiosis did not disrupt their ability to progress postnatally. Collectively, these findings suggest that a new stage for where mechanistic insights into the origin of meiotic competence in the male germline should be explored. Moreover, the findings place further precedence on defining how outside exposures can disrupt programming at the earliest stages of male germ cell development that will manifest at advanced maturation stages and lead to genomic abnormalities.

## 1 Introduction

The role of meiosis to reduce chromosomal content by half, thereby producing haploid gametes, is fundamental for all sexually reproducing organisms (Handel and Schimenti 2010). Impairment of the process leads to aneuploidy or arrest of germ cell maturation that ultimately manifests as sterility. In mammals, the trajectory of meiotic progression is different between males and females. In females, germ cells transition shortly after sex determination in embryogenesis from mitotic divisions to a first meiotic division, becoming arrested in prophase I as primary oocytes (Handel and Schimenti 2010). In contrast, meiosis in male germ cells does not initiate until well after birth and following a series of mitotic divisions as spermatogonia before becoming primary spermatocytes (Griswold 2016). While the process of meiosis has been well studied in both male and female germ cells, the mode by which programming for meiotic competence is established is undefined.

Spermatogonia are the foundation for continuity and robustness of the male germline during postnatal life (Oatley and Brinster 2008). The heterogeneous population can be broadly classified as undifferentiated and differentiating subtypes. The undifferentiated subpopulation is comprised of a spermatogonial stem cell (SSC) pool and abundant progenitor spermatogonia that transiently amplifies in number via mitotic divisions before transitioning to the differentiating state in response to a periodic pulse of retinoic acid. The differentiating spermatogonia undergo another series of ordered mitotic divisions before transitioning to meiotic divisions as primary and then secondary spermatocytes.

The spermatogonial population becomes established during postnatal development from a prospermatogonial precursor population that arose after sex determination in fetal development (Law et al. 2019; Hilscher et al. 1974). Recently, developmental kinetics of the prospermatogonial to postnatal spermatogonial transition was described in the mouse model (Law et al. 2019). Yet, the mode by which programming for meiotic competence is established in the male germ cell lineage remains undefined.

In a previous study, we engineered a transgenic mouse line for conditional overexpression of the transcriptional regulator inhibitor of DNA binding 4 (*Id4*), designated *Id4* ^*cOE*^, and discovered that constitutive expression in male germ cells beginning at the prospermatogonial stage dramatically impairs spermatogenesis (Helsel et al. 2017). At postnatal day (P) 0, the number of prospermatogonia in testes of *Id4* ^*cOE*^ mice is not different compared to wild‐type control mice demonstrating that neither proliferation nor survival is significantly altered. In addition, by P10 seminiferous tubules of *Id4* ^*cOE*^ mice lack differentiating spermatogonia indicating that transition from a progenitor state is impaired. Interestingly, although sperm are never produced, at 2 months of age arrested spermatocytes are observable in *Id4* ^*cOE*^ seminiferous tubules. Here, we used this *Id4* ^*cOE*^ model to investigate the impacts of conditional overexpression beginning at either the prospermatogonial stage of spermatogenic lineage development or in differentiating spermatogonia during postnatal life on the development of spermatocytes and meiotic progression. Outcomes revealed that activation of *Id4* overexpression in prospermatogonia during fetal development results in the formation of spermatocytes in postnatal life, but meiosis is abnormal, resulting in spermatogenic arrest. In contrast, spermatocytes generated when *Id4* overexpression is activated in differentiating spermatogonia during postnatal life complete meiosis and yield spermatozoa. In addition, activation of *Id4* overexpression in female germ cells during fetal development does not inhibit initiation or completion of meiosis. Together, these findings indicate that the programming for meiosis in the male germline is established during the prospermatogonial phase of development.

## 2 Materials and Methods

### 2.1 Animals

To generate mice with aberrant expression of *Id4* in prospermatogonia, differentiating spermatogonia, or oocytes females containing a *floxed‐Stop‐Id4* overexpression transgene in the *Rosa26* locus (*Id4* ^*cOE*^) were mated with males possessing different *Cre* expressing transgenes. Creation and validation of the *Id4* ^*cOE*^ model was described in previous studies (Helsel et al. 2017). The *Ddx4‐Cre* and *Stra8‐Cre* transgenic mouse lines were generated and validated in previous studies (Sadate‐Ngatchou et al. 2008; Gallardo et al. 2007). All animal procedures were approved by the Washington State University Institutional Animal Care and Use Committee (IACUC protocol #6731).

### 2.2 Histology

Testes and ovaries were excised from mice following euthanasia and fixed in Bouin's solution for 8 h or 4% paraformaldehyde for 2 h, respectively. Following dehydration, tissue samples were embedded in paraffin and 4 µm sections created using a microtome (Leica RM2235, Mannheim, Germany). Sections were then dewaxed, rehydrated, and processed for hematoxylin and eosin (H&E) or periodic acid‐Schiff (PAS) staining.

### 2.3 Mating Trials

Pubertal *Id4* ^*cOE*^ females were mated with *Ddx4* ^*Cre*^ or *Stra8* ^*Cre*^ males to generate *Id4* ^*cOE*^ *;Ddx4* ^*Cre*^ males (*Id4* ^*ProSpgOE*^), *Id4* ^*cOE*^ *;Stra8* ^*Cre*^ (*Id4* ^*DiffSpgOE*^) males or *Id4* ^*cOE*^ *;Ddx4* ^*Cre*^ females (*Id4* ^*OocyteOE*^). Fertility assessment of all *Id4* ^*cOE*^ mouse models and control counterparts was conducted at 2–4 months of age by paring with wild‐type females or males. The number of pups produced by each pairing over a 3‐month period was recorded.

### 2.4 Imaging

Testis cross‐sections and immunostained chromosome spreads were examined under microscopy using an ECLIPSE E200 (Nikon, Tokyo, Japan) or Leica TCS SP5 (Mannheim, Germany) platform. Images were captured by a Charge Coupled Device (CCD) digital camera using cellSens (Ver.2.2) acquisition software (Olympus).

### 2.5 Chromosome Spread Analysis

Chromosome spreads of spermatocytes from 21‐day‐old control, *Id4* ^*ProSpgOE*^, and *Id4* ^*DiffSpgOE*^ mice were performed to assess stages of meiotic prophase. Briefly, seminiferous tubules were transferred to a hypotonic buffer (30 mM Tris‐HCl at pH 8.2, 17 mM sodium citrate, 5 mM ethylenediaminetetraacetic acid, 50 mM sucrose, 5 mM dithiothreitol, and 0.5 mM phenylmethylsulfonyl fluoride) for 30 min at room temperature. Sucrose (100 mM) was dropped onto a clean slide. Seminiferous tubules were mixed with the sucrose drop on the clean slide and then disrupted to obtain a cell suspension, which was then spread over the slide immersed in 1% PFA. Adhesive slides with the cell suspension were put into a chamber with hot water (90°C–100°C) at normal atmospheric pressure overnight at RT. Slides were then washed in Antibody Dilution Buffer (ADB) (0.1% cold fish skin gelatin, 0.5% Triton X‐100, and 1% bovine serum albumin [BSA] in PBS) for 1 h the following day. A combination of primary antibodies (Supporting Information S1: Table S1) that had been diluted in ADB and incubated overnight at 37°C in a wet chamber was added. After being washed with ADB for 30 min, the slides were washed with ADB for 90 min and incubated with secondary antibodies overnight at 37°C. The slides were then washed in ADB for 30 min and PBS for 1 h. Slides were exposed to Hoechst (H33342) for 1 min and mounted in 50% glycerol before being examined under a Leica TCS SP5 microscope (Leica, Mannheim, Germany).

### 2.6 Quantification of Meiotic Prophase Distribution

Quantitative comparison was made for the distribution of spermatocytes at different stages of meiotic prophase in testes of adult male mice with conditional overexpression of *Id4*, initiated at either the prospermatogonial (*Id4* ^*ProSpgOE*^) or differentiating spermatogonial (*Id4* ^*DiffSpgOE*^) stage of development and in control mice without *Id4* overexpression. The percentage of different stages of meiotic prophase was quantified in 200 spermatocytes from three different males of each genotype.

### 2.7 Quantification of MLH1 Foci

Quantitative comparison was made of the MLH1 foci in testes of male mice between *Id4* ^*ProSpgOE*^ and control mice. The number of MLH1 foci on each chromosome was analyzed in 240 pachytene spermatocytes from three different males of each genotype.

### 2.8 Statistical Analysis

All quantitative data are presented as mean ± standard error of mean (SEM) for at least three biological replicates. Differences between means were examined using the general linear‐model ANOVA with multiple comparisons or *t*‐test function of GraphPad Prism 5 (La Jolla, CA, USA). Differences between means were considered significant when *p* < 0.05.

## 3 Results

### 3.1 Conditional Overexpression of *Id4* Beginning at the Prospermatogonial Phase of Male Germline Development Leads to Meiotic Arrest

**Figure 1:**
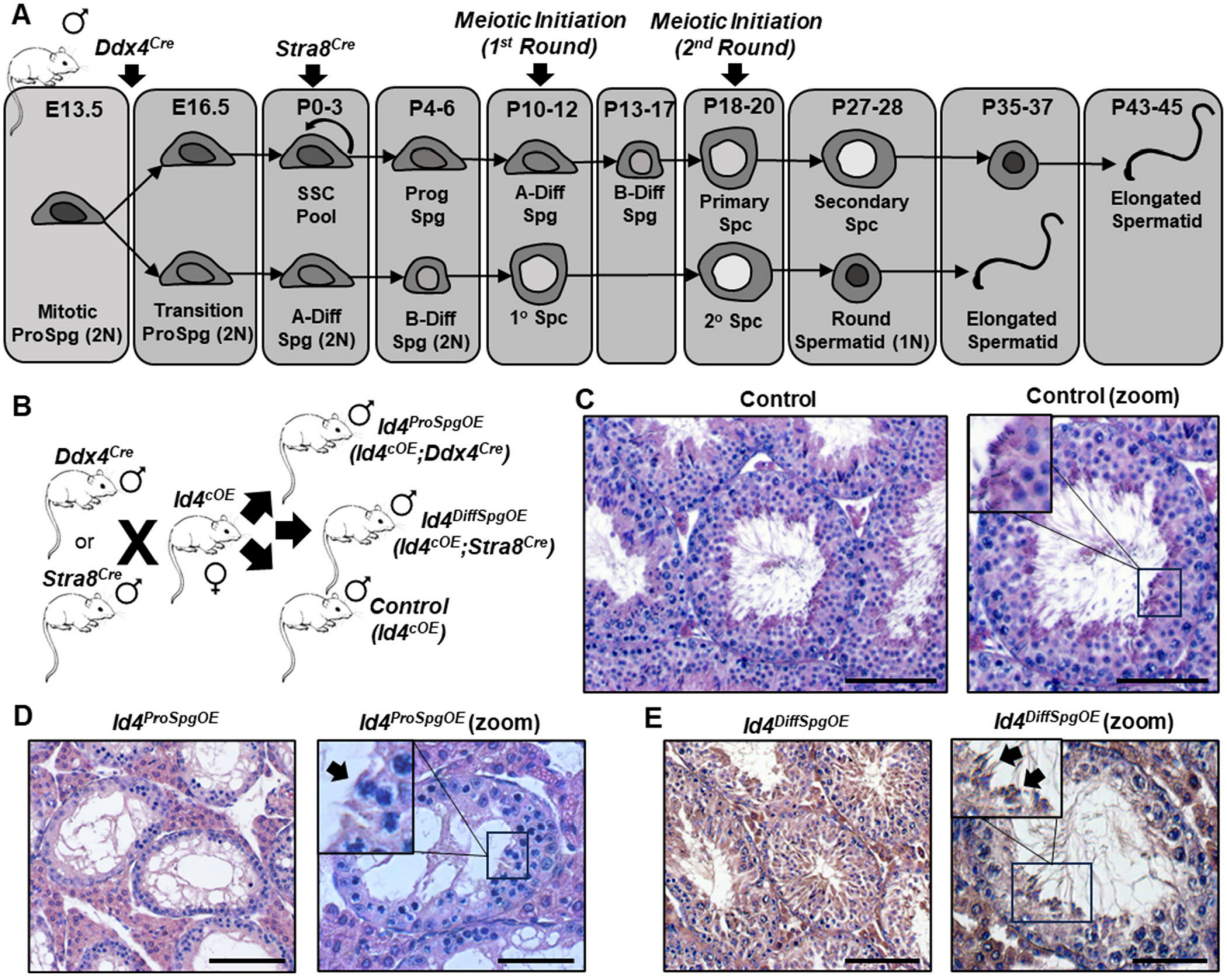
Impacts of conditional overexpression of *Id4* in the male germline at various stages of development on spermatogenesis in postnatal life. (A) Schematic of spermatogenic lineage establishment in the mouse. The first and second, as well as subsequent, rounds of spermatogenesis arise from postnatal spermatogonial populations that derived from prospermatogonial precursors. Regardless of the round of spermatogenesis, transition from the type B differentiating spermatogonia (B‐Diff) to primary spermatocyte (Spc) stage signifies the initiation of mitotic prophase. *Cre* recombinase expression driven by the *Ddx4* or *Stra8* promoter allows for creating mouse models with conditional overexpression of transgenes specifically in prospermatogonia or differentiating spermatogonia, respectively. (B) Schematic of mouse models generated to conditionally overexpress *Id4* beginning at the prospermatogonial or differentiating spermatogonial stages of spermatogenesis. (C–E) Representative images of cross‐sections from testes of 2‐month‐old control mice (C), mice with conditional overexpression of *Id4* beginning at the prospermatogonial stage of development (D), and mice with conditional overexpression of *Id4* beginning at the differentiating spermatogonial stage of development (E). Arrow in (D) denotes an arrested spermatocyte and arrows in (E) denote elongating spermatids. Bars are 100 and 50 μm (zoom images).

In mice, and possibly all mammals, the first round of spermatogenesis derives from a subset of prospermatogonia that transition directly to differentiating spermatogonia shortly after birth, and all subsequent rounds of spermatogenesis derive from an SSC/progenitor pool that arose from another subset of prospermatogonia (Figure 1A) (Law et al. 2019; Yoshida et al. 2004). In male mice with constitutive expression of *Id4* beginning at the prospermatogonial stage (designated hereafter as *Id4* ^*ProSpgOE*^), generated by crossing *Id4* ^*cOE*^ and *Ddx4* ^*Cre*^ transgenic mice (Figure 1B), we discovered that the SSC to progenitor spermatogonial transition in the second round of spermatogenesis is blocked leading to sterility (Helsel et al. 2017). However, unlike the case in wild‐type mice (Figure 1C), the first round of spermatogenesis initiates with formation of primary spermatocytes but haploid round spermatids do not arise in *Id4* ^*ProSpgOE*^ males (Figure 1D and Supporting Information S1: Figure S1), and the mice do not sire offspring although mating occurs which confirms that spermatozoa are not produced (Helsel et al. 2017). With advancing adult age, the germline becomes ablated in *Id4* ^*ProSpgOE*^ males leading to a Sertoli cell‐only phenotype by 6 months (Supporting Information S1: Figure S1). These observations suggest that in addition to impaired development of the undifferentiated spermatogonial population, meiotic progression is disrupted in an *Id4* overexpression state and the SSC pool is either not established or exhausted.

### 3.2 Conditional Overexpression of *Id4* Beginning at the Differentiating Spermatogonial Step of Spermatogenesis Does Not Cause Meiotic Arrest

Based on the observed phenotype of *Id4* ^*ProSpgOE*^, we postulated that meiotic arrest was caused by aberrant *Id4* expression in spermatocytes, interfering with the process of chromosome recombination. To test this, a second mouse model was generated with conditional activation of *Id4* overexpression in differentiating spermatogonia (Figure 1B), which form in postnatal life and precede the initiation of meiotic prophase, by crossing *Id4* ^*cOE*^ and *Stra8* ^*Cre*^ mice (designated hereafter as *Id4* ^*DiffSpgOE*^). The *Stra8* ^*Cre*^ transgene is first activated in differentiating spermatogonia around postnatal day 4 in male mice (Sadate‐Ngatchou et al. 2008), thus any forming spermatocytes would possess aberrant *Id4* expression analogous to those in *Id4* ^*ProSpgOE*^ mice. Like the *Id4* ^*ProSpgOE*^ mice, adult *Id4* ^*DiffSpgOE*^ males (*n* = 3 different mice) did not sire offspring in a multi‐month mating trial with wild‐type females (Supporting Information S1: Figure S2A). Low amounts of sperm were observed in epididymal flushing from adult (> 2 months of age) *Id4* ^*DiffSpgOE*^ mice and examination of cross‐sections from testes revealed impaired spermatogenesis but qualitatively the spermatogenic lineage was intact with the presence of germ cells at all stages of maturation (Figure 1E and Supporting Information S1: Figure S2C). Importantly, both primary and secondary spermatocytes were present as well as round and elongate spermatids. Visually reduced spermatogenesis was observed in > 90% of seminiferous tubule cross‐sections, thus a low efficiency of Cre‐mediated recombination of the conditional overexpression transgene was not an overriding cause of the phenotype in *Id4* ^*DiffSpgOE*^.

### 3.3 Abnormal Meiotic Progression in Spermatocytes Produced From Germ Cells With Conditional *Id4* Overexpression at the Prospermatogonial Phase

**Figure 2:**
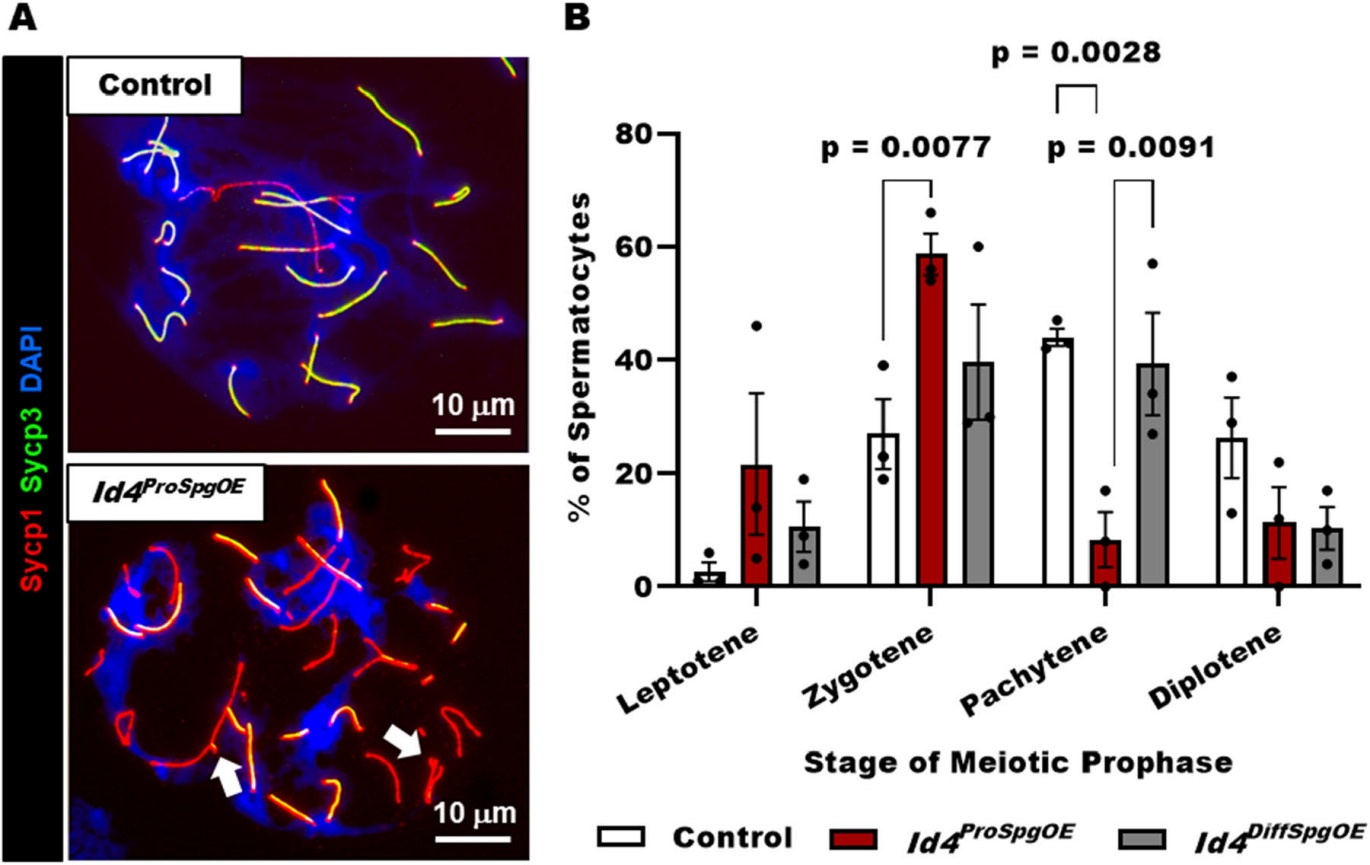
Conditional overexpression of *Id4* beginning at the fetal prospermatogonial stage of development impairs meiotic progression in postnatal spermatocytes. (A) Representative images of chromosome spreads from spermatocytes of 21‐day‐old control and *Id4* ^*ProSpgOE*^ mice. Synaptonemal complexes are immunostained for the proteins Sycp1 and Sycp3 and DNA is labeled with DAPI. Arrows denote bubbles in the synapsed chromosomes. Bars are 10 μm. (B) Quantitative comparison of the distribution of spermatocytes at different stages of meiotic prophase in testes of adult male mice with conditional overexpression of *Id4* beginning at the prospermatogonial (*Id4* ^*ProSpgOE*^) or differentiating spermatogonial (*Id4* ^*DiffSpgOE*^) stage of development and control mice without *Id4* overexpression. Data are mean ± SEM for *n* = 3 different males of each genotype, and *p*‐values for statistical significance are included for comparisons that are different.

**Figure 3:**
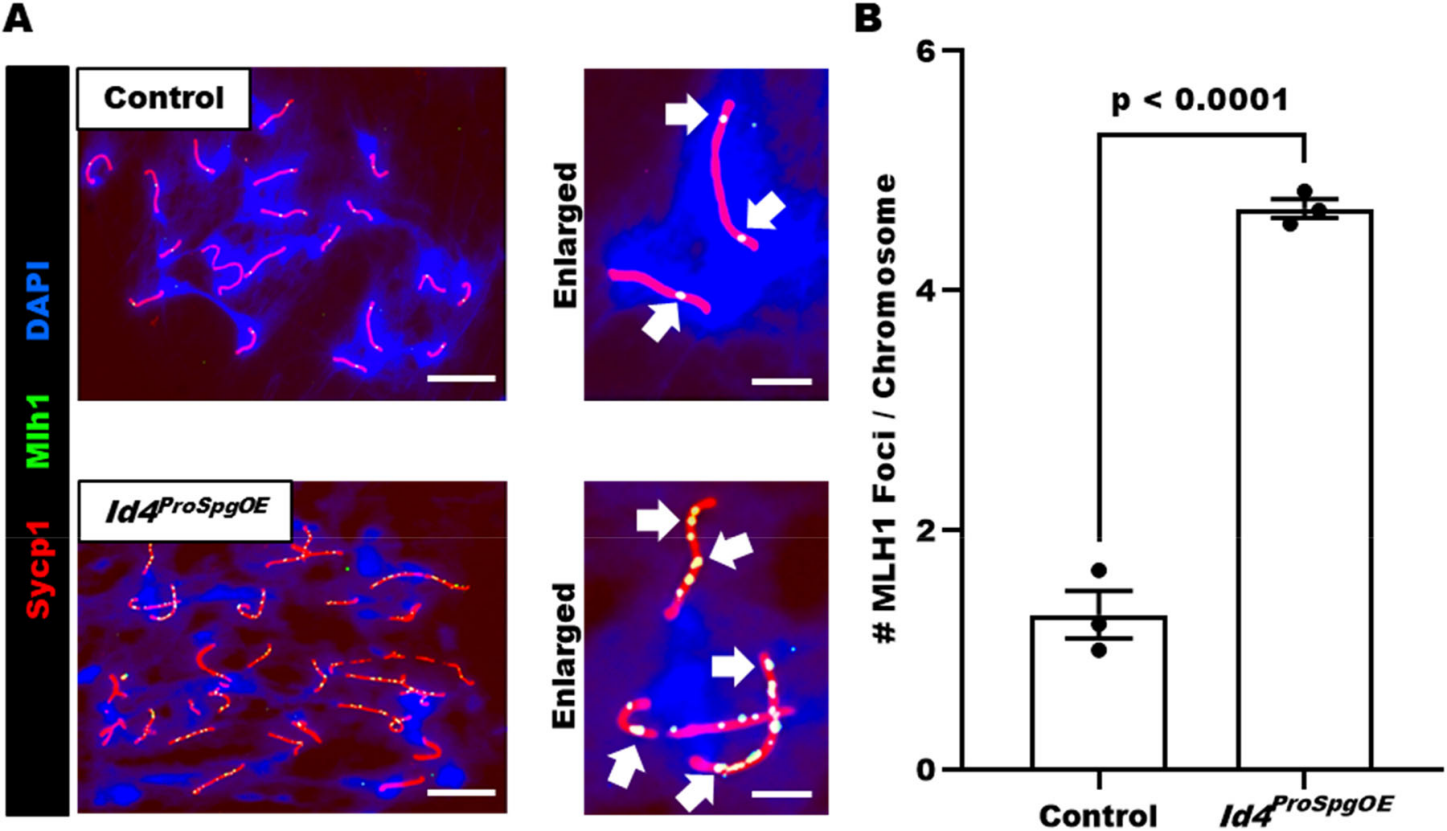
Abnormal DNA double stand break formation in postnatal spermatocytes derived from prospermatogonia with conditional overexpression of *Id4*. (A) Representative images of pachytene spermatocyte chromosome spreads stained for Mlh1 foci (denoted by arrows) that form at DNA double strand breaks during meiotic prophase. Bars are 10 and 3 μm (enlarged images). (B) Quantitative comparison of the number of MLH1 foci between control and *Id4* ^*ProSpgOE*^ mice. Data are mean ± SEM for *n* = 3 different males of each genotype, and a *p*‐value for statistical analysis is included.

To explore further the spermatocyte arrest in *Id4* ^*ProSpgOE*^ mice, chromosome spread analysis was conducted with spermatocytes isolated from 21‐day old *Id4* ^*ProSpgOE*^ and control mice that possessed the *Id4* conditional overexpression transgene but lacked a *Cre* transgene. We examined meiotic prophase I during which DNA double stand breaks (DSBs) occur in primary spermatocytes and homologous chromosomes synapse and recombine. To visualize the process, immunofluorescent staining for Sycp1 and Sycp3 which are components of the synaptonemal complex (SC) was used. In meiocytes from *Id4* ^*ProSpgOE*^, several abnormalities were observed, including asynapsis of homologous chromosomes and formation of forks and bubbles in synapsed chromosomes (Figure 2A and Supporting Information S1: Figure S3). Next, we aimed to determine whether progression through prophase was altered in the spermatocyte population of *Id4* ^*ProSpgOE*^ mice. The mean (±SEM) percentage of cells in leptotene, zygotene, pachytene, and diplotene phases during normal conditions in control mice was measured to be 2.7 ± 1.6%, 27.7 ± 6.1%, 44.0 ± 1.5%, and 26.3 ± 7.1%, respectively (*n* = 3 different males). In comparison, the percentage of cells for *Id4* ^*ProSpgOE*^ mice (*n* = 3 different males) in zygotene (58.7 ± 3.7%) and pachytene (8.3 ± 4.9%) phases was measured to be significantly different than control, whereas the percentage of cells in leptotene (21.7 ± 12.4%) and diplotene (11.3 ± 6.4%) phases were not different (Figure 2B). In contrast, chromosome spread analysis of the spermatocyte population in 21‐day old *Id4* ^*DiffSpgOE*^ mice revealed that distribution of cells in different stages of meiotic prophase was not different than control males (Figure 2B), implying that aberrant expression of *Id4* in differentiating spermatogonia leading up to or during the spermatocyte stage of germ cell maturation does not disrupt progression through meiosis. In addition, based on immunostaining for Mlh1, which accumulates at sites of DNA damage, we observed an elevated number of foci in pachytene stage spermatocytes from *Id4* ^*ProSpgOE*^ mice compared to controls (Figure 3A). Compared to pachytene spermatocytes of control mice in which 1.3 ± 0.2 (mean ± SEM for *n* = 3 different mice) Mlh1 foci were measured per chromosome, chromosomes in cells from *Id4* ^*ProSpgOE*^ mice were found to have 4.7 ± 0.1 (mean ± SEM for *n* = 3 different mice) Mlh1 foci (Figure 3B), suggesting that the molecular machinery governing genesis and repair of DSBs during meiotic prophase is impaired in spermatocytes that were derived from precursor prospermatogonia with aberrant expression of *Id4*.

### 3.4 Conditional Activation of *Id4* Overexpression Beginning at the Oogonial Phase of Female Germline Development Does Not Lead to Impaired Meiosis

**Figure 4:**
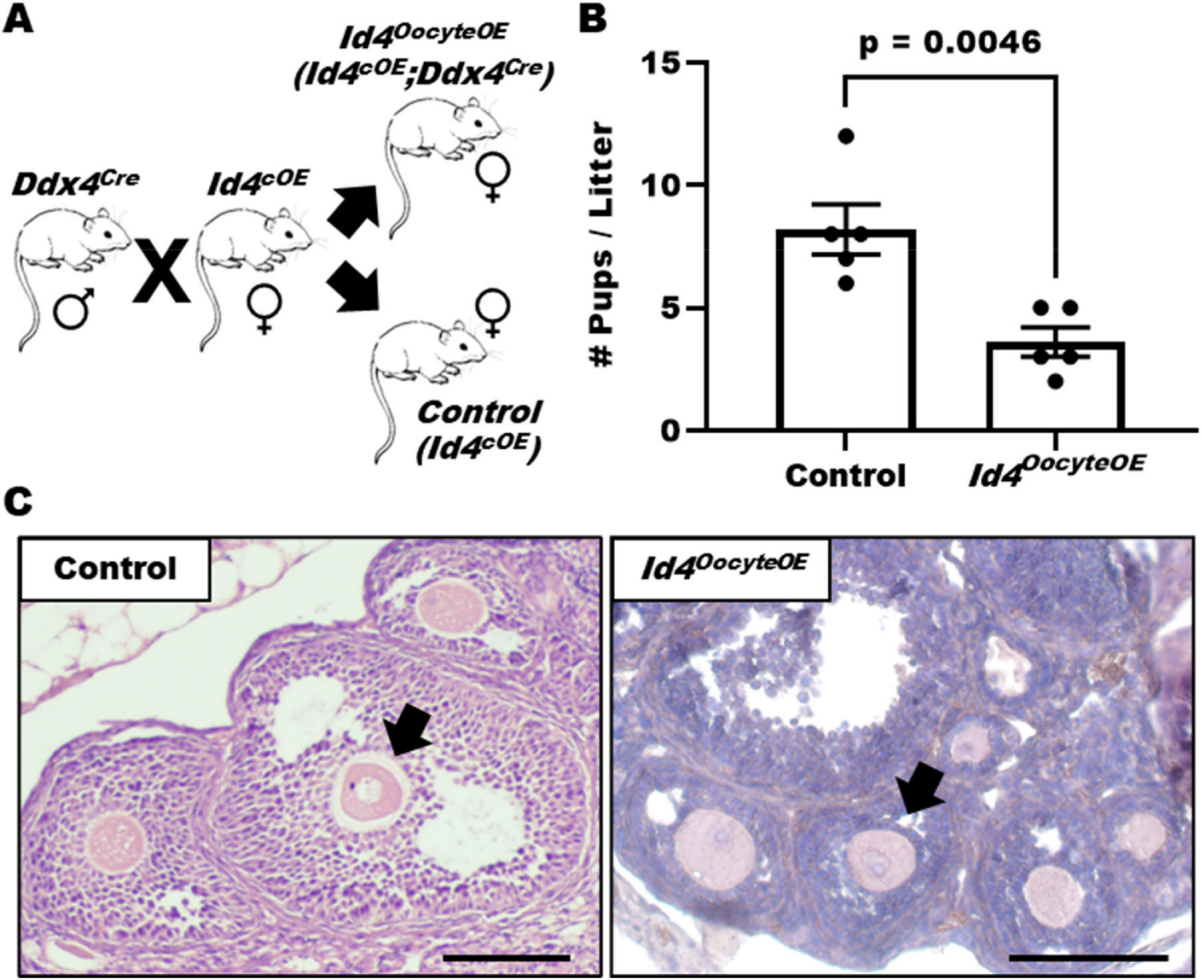
Conditional overexpression of *Id4* in oocytes beginning at the fetal stage of development does not impair meiotic progression. (A) Schematic of the mouse model generated to conditionally overexpress *Id4* in oocytes beginning in fetal development. (B) Quantitative comparison of fecundity between adult female mice with (*Id4* ^*OocyteOE*^) or without (control) conditional overexpression of *Id4* in oocytes. Data are mean ± SEM for *n* = 3 different females of each genotype, and a *p*‐value for statistical analysis is included. (C) Representative images of cross‐sections from the ovaries of adult control and *Id4* ^*OocyteOE*^ mice. Developing follicles (indicated by arrows) can be observed from both genotypes. Bars are 50 μm.

As an additional assessment of the impact of aberrant *Id4* expression on meiotic progression in germ cells, the phenotype of female mice with conditional overexpression in oocytes was explored. To achieve this, we utilized female littermates of *Id4* ^*ProSpgOE*^ male mice in which constitutive *Id4* expression was activated in oocytes that normally arrest at the leptotene stage of prophase I during fetal development, designated as *Id4* ^*OocyteOE*^ (Figure 4A). Outcomes of a 3‐month mating trial revealed that adult female *Id4* conditional overexpression mice (*n* = 3 different females) were fertile, although the number of pups born per litter was reduced compared to control females (Figure 4B). Examination of uteri from pregnant *Id4* overexpression females revealed several fetuses in the process of re‐absorption, which was not observed with control females, suggesting that the cause of reduced litter size was elevated lethality of fetuses that inherited the activated *Id4* overexpression transgene. Lastly, we examined cross‐sections of ovaries from conditional overexpression and control mice to assess folliculogenesis. As expected from the fertility status, follicles at various stages of maturation were observed in cross‐sections from both overexpression and control females (Figure 4C). We observed primary, secondary, and tertiary follicles, as well as corpa lutea. Taken together, these findings demonstrate that, like spermatocytes derived from differentiating spermatogonia with *Id4* overexpression, aberrant *Id4* levels in oocytes does not prevent meiotic progression in female germ cells.

## 4 Discussion

Meiosis in germ cells is a fundamental process for all sexually reproducing organisms, but the molecular programming that directs it is undefined, especially in mammalian cells. In both male and female germ cells, a switch from mitosis to meiosis is key to produce genetically unique haploid gametes. Previous studies have established that activation of retinoic acid signaling initiates the process (Griswold 2016; Anderson et al. 2008; Koubova et al. 2006), in part through downregulation of *Dmrt1* expression (Minkina et al. 2014). Indeed, impairment of retinoic acid biosynthesis in the gonad or signaling in germ cells abolishes the initiation of meiosis (Velte et al. 2019; Endo et al. 2015; Hogarth et al. 2011). In addition, *Dmrt1* deficiency leads to premature initiation of meiosis (Minkina et al. 2014; Matson et al. 2010). Although these findings have shed light on how it is initiated and a multitude of other studies have yielded information about the nuts and bolts of meiosis, the programming that drives the progression of the process is not well understood.

In mammals, the timeframe of male spermatogenic lineage establishment is post sex determination in fetal development through to the first appearance of elongated spermatids in the seminiferous epithelium during postnatal life. This process initiates with the derivation of prospermatogonia from primordial germ cells at sex determination in the fetal gonad (Hilscher et al. 1974; McLaren 1995). The prospermatogonia then undergo a period of mitotic divisions followed by arrest. During the neonatal period, prospermatogonial mitosis resumes and transition to postnatal spermatogonial subtypes occurs including formation of a stem cell pool (Law et al. 2019). Differentiating spermatogonia arise in the prepubertal period and initiate a first meiotic division to form primary spermatocytes. Following completion of meiosis I, secondary spermatocytes arise that undergo a second meiotic division to yield haploid spermatids that undergo spermiogenesis to form elongated spermatids. The step in this timeline of development that programming for meiotic competence is established has not been defined.

Previous studies with mice have revealed that retinoic acid signaling in postnatal spermatogonia triggers eventual initiation of meiotic prophase (Griswold 2016; Agrimson et al. 2016; Hogarth 2015), even when the cells are precociously exposed to the inducer during prepubertal development (Velte et al. 2019; Johnson 2023). Thus, the programming for meiotic competence is established during the perinatal period, which could be in the prospermatogonial or initial spermatogonial subtype phases. A recent study reported that delay in the timing of when prospermatogonia undergo mitotic arrest during late fetal development results in loss of tight control on the expression of genes needed for meiosis, which normally occurs in differentiating spermatogonia (Du et al. 2021). In the present study, the phenotype of *Id4* ^*DiffSpgOE*^ male mice demonstrates that *Id4* overexpression in differentiating spermatogonia and primary spermatocytes per se does not block either the initiation or progression of meiosis. However, because Mlh1 foci analyses was not performed with *Id4* ^*DiffSpgOE*^ spermatocytes whether increased DNA damage occurs is undefined. In addition, the phenotype of mutant female mice produced in this study corroborators the conclusion that progression of meiosis can occur in the presence of *Id4* overexpression. Although we did not directly assess meiotic normalcy or the possibility of increased DNA damage in oocytes of female mice with *Id4* overexpression initiating during fetal development, the fact that they were able to produce viable offspring suggests normal progression and development. In contrast, meiotic progression is halted in spermatocytes that are descended from prospermatogonia in which aberrant *Id4* expression was initiated.

How *Id4* overexpression beginning at the prospermatogonial stage impacts meiotic progression at a later stage of development is undefined. Id family proteins are known dominant‐negative regulators of other basic helix‐loop‐helix (bHLH) transcription factors. Thus, it is plausible to speculate that overexpression of *Id4* at a stage in male germline development when the gene is ordinarily not active leads to impaired action of bHLH molecules that establish the molecular framework for future meiotic initiation and progression. Taken together with findings of previous studies, results of the present study suggest that the prospermatogonial mitotic arrest period is when programming for meiotic competence is established during the trajectory of spermatogenic lineage establishment.

## Author Contributions

Qi‐En Yang conceived and performed the research and analyzed the data. Melissa J. Oatley performed the research and analyzed the data. Mingyao Yang performed the research and revised the manuscript. Jon M. Oatley conceived the research, analyzed the data, secured funding for the research, and wrote the manuscript.

## Conflicts of Interest

The authors declare no conflicts of interest.

## Supporting information

Supplementary‐Figures‐Rev1.

## Data Availability

The data that support the findings of this study are available from the corresponding author upon reasonable request.
