# Supplementary material for: Programming for Meiotic Competence in Mouse Male Germ Cells is Established at the Perinatal Precursor Stage of Development
Source: Mol Reprod Dev. 2025 May 25;92(5):e70032. doi: 10.1002/mrd.70032 (PMC12104550; doi:10.1002/mrd.70032)
Supplement: Supplementary file 1 — Supplementary‐Figures‐Rev1. [file MRD-92-e70032-s001.docx]

**
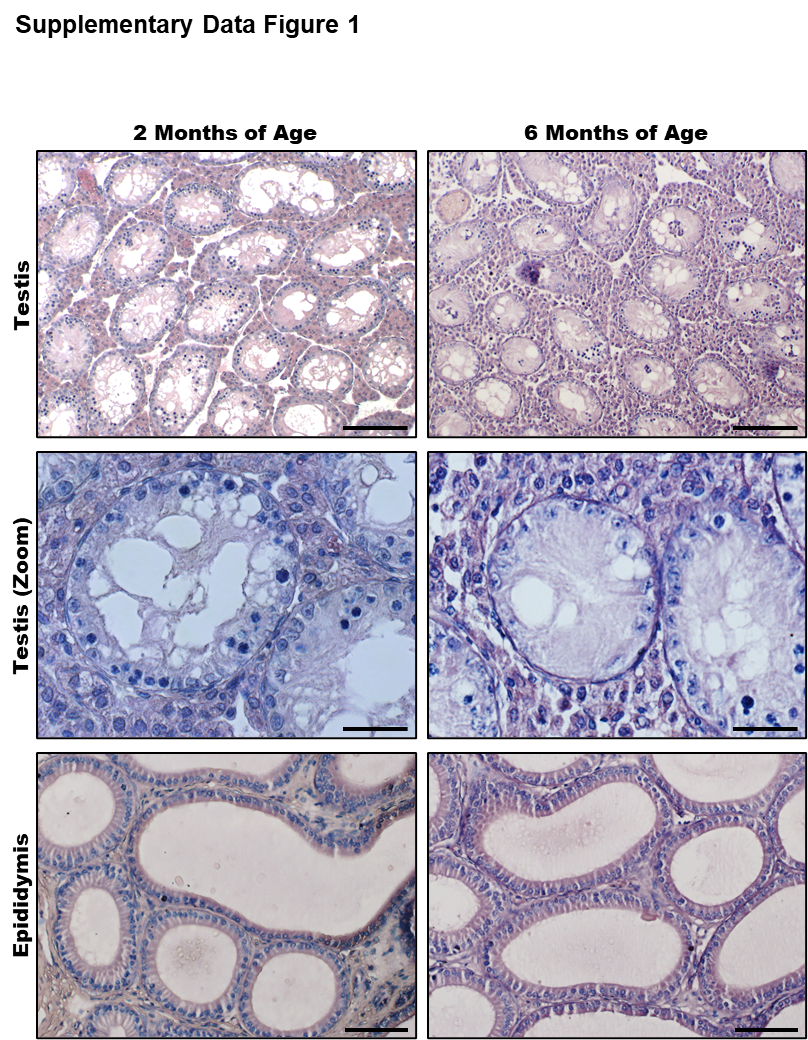
**

**Supplementary Figure S1. Impact of conditional overexpression of *Id4* at the prospermatogonial stage of male germline development.** Representative images of cross-sections from testes and epididymis of *Id4^ProSpgOE^* mice at 2 and 6 months of age. Complete block of spermatogenesis can be clearly observed at both ages with the most advanced germ cell being primary spermatocytes at 2 months of age. Bars are 100 μm or 50 μm (zoom testis images and epididymis images).

**
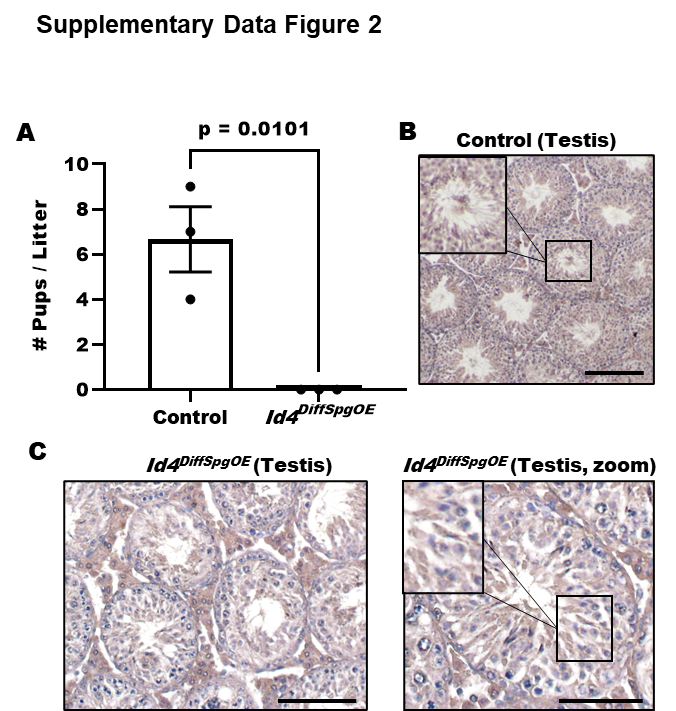
**

**Supplementary Figure S2. Impact of conditional overexpression of *Id4* at the differentiating spermatogonial stage of male germline development.** (**A**) Fertility assessment of *Id4^DiffSpgOE^* males at 2-4 months of age compared to control mice without *Id4* overexpression. Data are mean±SEM for n=3 different males of each genotype. None of the *Id4^DiffSpgOE^* males sired offspring during the mating trial period owing to severe reduction in sperm counts. (**B and C**)**,** Representative images of cross-sections from testes of control (**B**) and *Id4^DiffSpgOE^* (**C**) mice at 4 months of age. Complete spermatogenesis can be observed in both control and *Id4^DiffSpgOE^* mice as indicated by the presence of elongating spermatids. Bars are 100 μm and 50 μm (zoom image).


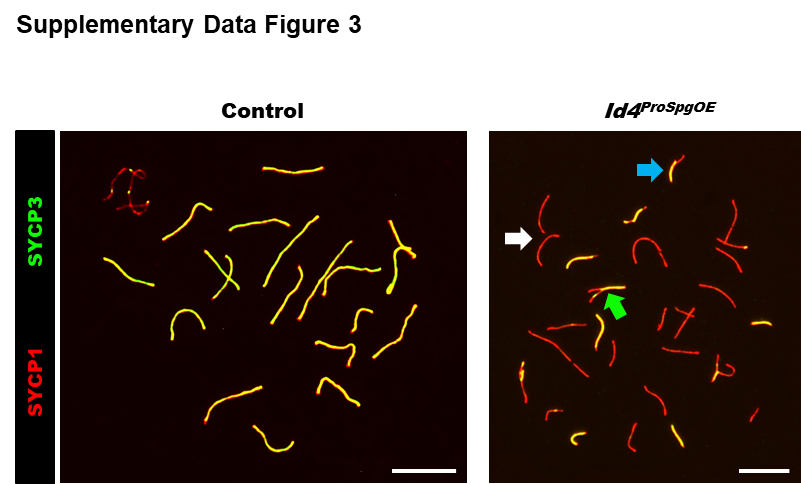


**Supplementary Figure S3.** Representative images of chromosome spreads for primary spermatocytes in male mice with conditional *Id4* overexpression beginning at the prospermatogonial stage of male germline development (*Id4^ProSpgOE^*) or without *Id4* overexpression (control). Several abnormalities of meiotic prophase can be observed in the *Id4^ProSpgOE^* spermatocyte chromosomes including asynapsis (white arrow), partial synapsis (blue arrow), and forks (green arrow). Bars are 10 μm.

| **Supplementary Table S1. List of antibodies used in this study.** | | | | |
| --- | --- | --- | --- | --- |
| Antibody | Source | Dilution | Source | Reference |
| SYCP1  SYCP3  MLH1 | Goat  Rabbit  Rabbit | 1:200  1:500  1:500 | Santa Cruz  Abcam  Abcam | Cat.sc-20837  Cat.ab15093  Cat.ab92312 |
| CoraLite-488-Conjugated Affinipure Anti-Rabbit IgG  CY3-Conjugated Affinipure Anti-Goat IgG | Donkey  Donkey | 1:500  1:500 | Proteintech  Proteintech | Cat.SA00013-6  Cat.SA00009-3 |
